# Multimodal Artificial Intelligence Using Endoscopic USG, CT, and MRI to Differentiate Between Serous and Mucinous Cystic Neoplasms

**DOI:** 10.7759/cureus.85547

**Published:** 2025-06-08

**Authors:** Katsushi Seza, Katsunobu Tawada, Akitoshi Kobayashi, Kazuyoshi Nakamura

**Affiliations:** 1 Gastroenterology, Chiba Medical Center, Chiba, JPN; 2 Gastroenterology, Chiba Kaihin Municipal Hospital, Chiba, JPN; 3 Gastroenterology, Funabashi Municipal Medical Center, Funabashi, JPN; 4 Gastroenterology, Chiba Cancer Center, Chiba, JPN

**Keywords:** artificial intelligence, diagnostic accuracy, endoscopic ultrasound, mucinous cystic neoplasm, multimodal imaging, pancreatic cystic neoplasms, serous cystic neoplasm

## Abstract

Introduction

Serous cystic neoplasms (SCN) and mucinous cystic neoplasms (MCN) often exhibit similar imaging features when evaluated with a single imaging modality. Differentiating between SCN and MCN typically necessitates the utilization of multiple imaging techniques, including computed tomography (CT), magnetic resonance imaging (MRI), and endoscopic ultrasonography (EUS). Recent research indicates that artificial intelligence (AI) can effectively distinguish between SCN and MCN using single-modal imaging. Despite these advancements, the diagnostic performance of AI has not yet reached an optimal level. This study compares the efficacy of AI in classifying SCN and MCN using multimodal imaging versus single-modal imaging. The objective was to assess the effectiveness of AI utilizing multimodal imaging with EUS, CT, and MRI to classify these two types of pancreatic cysts.

Methods

We retrospectively gathered data from 25 patients with surgically confirmed SCN and 24 patients with surgically confirmed MCN as part of a multicenter study. Imaging was conducted using four modalities: EUS, early-phase contrast-enhanced abdominal CT, T2-weighted MRI, and magnetic resonance pancreatography. Four images per modality were obtained for each tumor. Data augmentation techniques were utilized, resulting in a final dataset of 39,200 images per modality. An AI model with ResNet was employed to categorize the cysts as SCN or MCN, incorporating clinical features and combinations of imaging modalities (single, double, triple, and all four modalities). The classification outcomes were compared with those of five experienced gastroenterologists with over 10 years of experience. The comparison is based on three performance metrics: sensitivity, specificity, and accuracy.

Results

For AI utilizing a single imaging modality, the sensitivity, specificity, and accuracy were 87.0%, 92.7%, and 90.8%, respectively. Combining two imaging modalities improved the sensitivity, specificity, and accuracy to 95.3%, 95.1%, and 94.9%. With three modalities, AI achieved a sensitivity of 96.0%, a specificity of 99.0%, and an accuracy of 97.0%. Ultimately, employing all four imaging modalities resulted in AI achieving 98.0% sensitivity, 100% specificity, and 99.0% accuracy. In contrast, experts utilizing all four modalities attained a sensitivity of 78.0%, specificity of 82.0%, and accuracy of 81.0%. The AI models consistently outperformed the experts across all metrics. A continuous enhancement in performance was observed with each additional imaging modality, with AI utilizing three and four modalities significantly surpassing single-modal imaging AI.

Conclusion

AI utilizing multimodal imaging offers better performance compared to both single-modal imaging AI and experienced human experts in classifying SCN and MCN.

## Introduction

Pancreatic serous cystic neoplasms (SCNs) and mucinous cystic neoplasms (MCNs) are uncommon. SCN exhibits a low malignancy rate, while carcinoma prevalence in MCN ranges from 11% to 18% [1-4]. Thus, distinguishing between these two conditions is crucial. Despite sharing similar imaging characteristics on a single modality, differentiating between SCN and MCN can be challenging. The classification accuracies for distinguishing SCN and MCN using computed tomography (CT), magnetic resonance imaging (MRI), endoscopic ultrasonography (EUS), and EUS with fine needle aspiration (FNA) are 25-90%, 56-75%, 50-51%, and 79-82%, respectively [5-10]. EUS with FNA demonstrates relatively high accuracy; however, it may not be adequate for determining the need for surgery or conservative management. Moreover, issues such as peritoneal dissemination and gastric seeding have been reported [11]. Therefore, there is a need to differentiate between SCN and MCN accurately without resorting to invasive techniques.

Therefore, in clinical practice, distinguishing between SCN and MCN necessitates the use of multiple modalities, including CT, MRI, and EUS. Classifying each disease can still be challenging despite employing various modalities.

Recently, many reports have demonstrated high accuracy in distinguishing SCN and MCN using artificial intelligence (AI) with a single modality (conventional single-modal AI: C-Single). The accuracies of EUS, CT, and MRI were 83%, 70-93%, and 75-92%, respectively [12-16]. Despite outperforming human diagnosis, these results are not enough to determine the treatment plan.

AI using multiple modalities has been proposed in various fields; however, limited studies have explored its application in the medical domain [17-20]. Most studies focused on C-Single with clinical features (C-SingleCF). By incorporating different imaging modalities into the model, a more accurate diagnosis was achieved compared to human diagnosis alone in clinical settings. Our study aimed to assess the effectiveness of AI utilizing multimodal imaging (MI-AI) with EUS, CT, and MRI in distinguishing between SCN and MCN. There are some studies using multi-phase or multi-sequence of single modality [16, 21]. To the best of our knowledge, this is the first investigation of AI employing multimodal imaging for pancreatic disorders. We seek to comprehensively evaluate all combinations of the four modalities, aiming to reveal the distinct features of each and the synergistic effects of their combinations.

## Materials and methods

We retrospectively gathered data from 29 patients with SCN and 27 patients with MCN, as confirmed by surgical pathology in a multicenter study. For the analysis, we utilized images from four modalities: EUS, early-phase contrast-enhanced abdominal CT (ECECT), T2-weighted MRI (T2), and magnetic resonance pancreatography (MRP). Seven patients were excluded due to a lack of imaging modalities. Ultimately, 25 patients with SCN and 24 patients with MCN were enrolled in the study.

**Figure 1:**
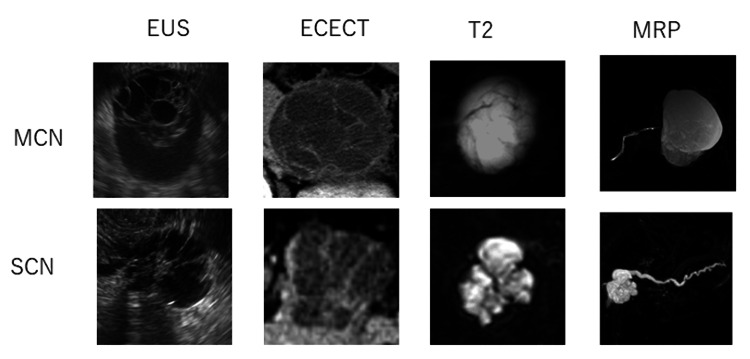
Input data for four modalities used in this study. MCN: mucinous cystic neoplasms, SCN: serous cystic neoplasms, EUS: endoscopic ultrasonography, ECECT: early phase of contrast-enhanced computed tomography, T2: T2-weighted magnetic resonance imaging, MRP: magnetic resonance pancreatography

On EUS, ECECT, and T2, all images were cropped to squares as the primary cystic lesion was centrally located. For MRP, images featuring the longest main pancreatic duct were chosen and cropped to squares. All images were converted into 256x256 pixels and converted to 8-bit grayscale. Four images were captured for each tumor using every imaging modality (Figure 1). Subsequently, the dataset was augmented, resulting in a total of 39,200 images for each imaging modality.

**Figure 2:**
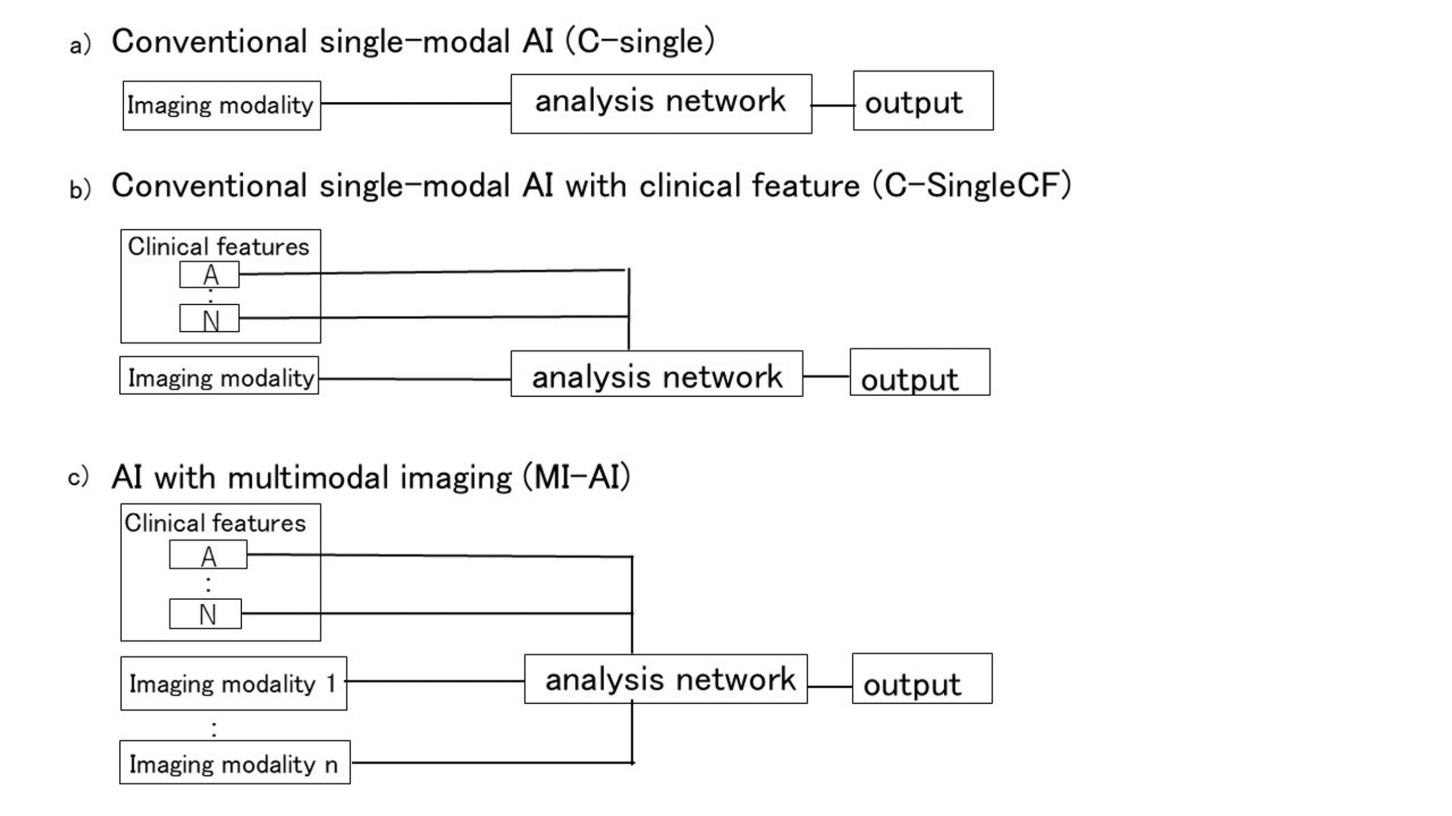
Workflow of single-modal and multimodal AI. MI-AI: AI utilizing multimodal imaging; C-SingleCF: C-Single with clinical features

**Figure 3:**
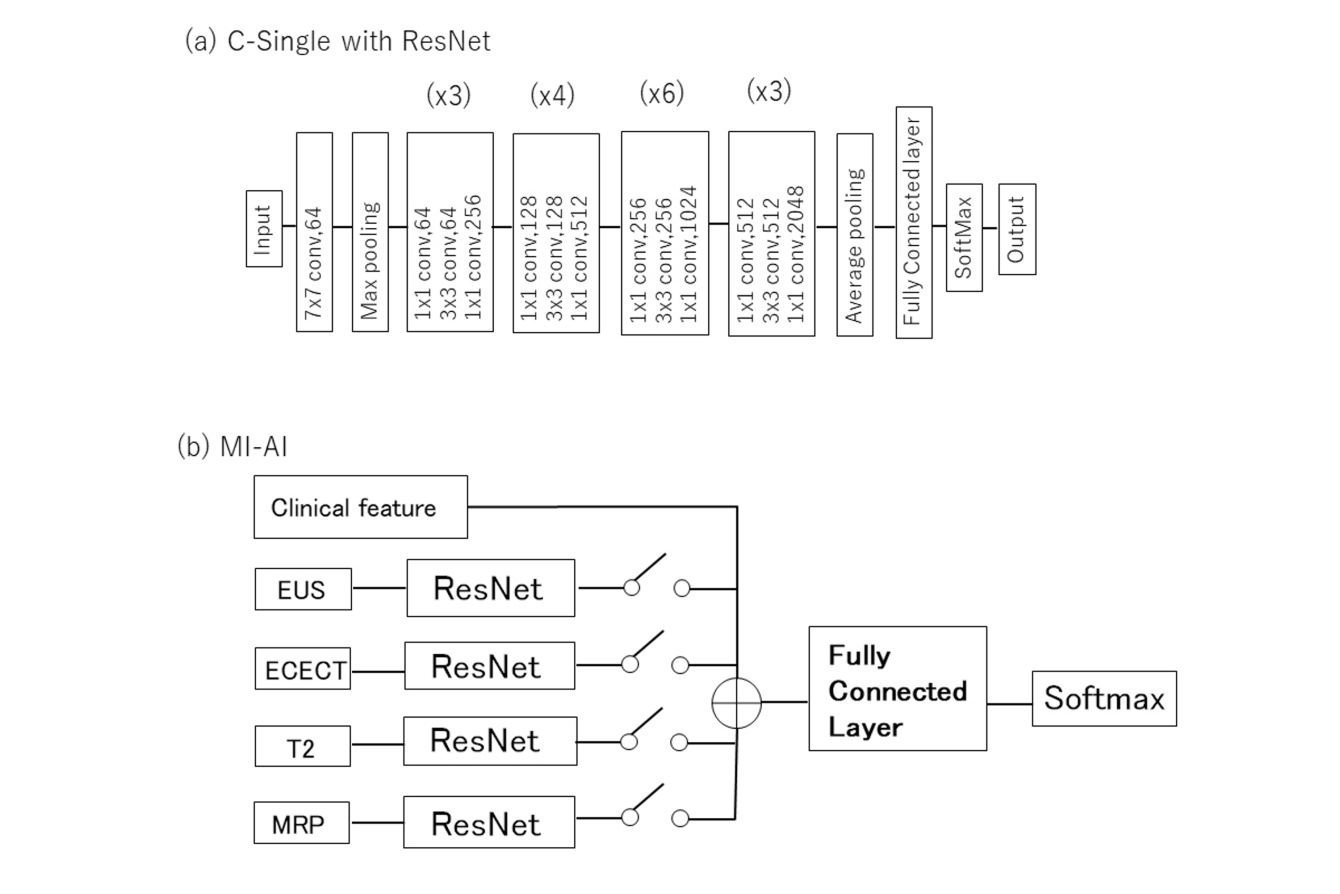
Workflows of (a) C-Single with ResNet and (b) MI-AI. C-Single: conventional single-modal artificial intelligence, ResNet: residual Network, conv: convolution,(xN): N times block repetition, EUS: endoscopic ultrasonography, ECECT: early phase of contrast-enhanced computed tomography, T2: T2-weighted magnetic resonance imaging, MRP: magnetic resonance pancreatography

Figure 2 presents an overview of the AI approaches and imaging modalities relevant to this study. We categorized the SCN and MCN using ResNet (Figure 3a) using a single imaging modality [13, 22, 23]. This AI model is equivalent to C-Single (Figure 2a). Figure 3b shows the workflow of MI-AI utilized in this study. The MI-AI inputs include clinical features and imaging modalities. Clinical features exhibiting statistical variances between SCN and MCN were chosen according to patient characteristics, such as sex, age, tumor size, location, and vascularity.

As shown in Fig. 3b, MI-AI with a single modality was comparable to C-SingleCF (Fig. 2b). MI-AI employing double, triple, and quad imaging modalities alongside clinical features were denoted as MI-Double, MI-Triple, and MI-Quad, respectively. These models were comparable to the model in Fig. 2c. The parameters are independently acquired across the layers of each modality. The final layer outputs from each modality are combined at the classifier stage and trained using a fully connected network.

Table 1 presents all 19 combinations of imaging modalities. C-Single AI utilizes one of the four modalities. C-SingleCF also utilizes a single modality from these four. MI-Double offers six ways to select two modalities from the four available. MI-Triple provides four ways to select three modalities from the four available. MI-Quad represents the single way of utilizing all modalities simultaneously.

**Table 1:** Combination of modalities and clinical information C-Single: conventional single-modal artificial intelligence; C-SingleCF: C-Single with clinical features; ECECT: early phase of contrast-enhanced computed tomography; EUS: endoscopic ultrasonography; MI-AI: artificial Intelligence utilizing multimodal imaging; MI-Double: MI-AI employing double imaging modalities with clinical feature; MI-Triple: MI-AI employing triple imaging modalities with clinical feature; MI-Quad: MI-AI employing quad imaging modalities with clinical feature; MRP magnetic resonance pancreatography; T2: T2-weighted magnetic resonance imaging

Model	Input	Clinical information	No. of imaging modalities
C-single	EUS	None	1
	ECECT	None	1
	T2	None	1
	MRP	None	1
C-SingleCF	EUS+SEX	Yes	1
	ECECT+SEX	Yes	1
	T2 +SEX	Yes	1
	MRP+SEX	Yes	1
MI-double	ECECT+MRP+SEX	Yes	2
	T2+MRP+SEX	Yes	2
	EUS+MRP+SEX	Yes	2
	ECECT+T2+SEX	Yes	2
	EUS+T2+SEX	Yes	2
	ECECT+EUS+SEX	Yes	2
MI-Triple	ECECT+T2+MRP	Yes	3
	EUS+T2+MRP+SEX	Yes	3
	EUS+T2+ECECT+SEX	Yes	3
	EUS+ECECT+MRP+SEX	Yes	3
MI-Quad	EUS+ECECT+T2+MRP	Yes	4
Experts	EUS	None	1
	ECECT	None	1
	T2	None	1
	MRP	None	1
	EUS+ECECT+T2+MRP+SEX	Yes	4

The classification results of AI were compared using three indicators: sensitivity, specificity, and accuracy, calculated through leave-one-out cross-validation.

CT, MRI, and EUS were individually assessed by five hepato-pancreato-biliary physicians (experts) with more than 10 years of experience in conducting EUS and imaging diagnosis. Subsequently, they reviewed a composite of all modalities. The outcomes from the experts and AI were then compared.

Continuous variables are reported as means and standard deviations. Student’s t-test, McNemar's test, and Fisher's exact test were employed to assess the statistical differences between continuous and discrete variables. Statistical significance was defined at P < 0.05.

## Results

Table 2 displays the characteristics of patients with SCN and MCN. The location of the tumor, vascularity, and sex exhibit statistically significant differences. Tumor vascularity was presented to the AI as images rather than 'Hyper' or 'Hypo' classifications to eliminate human bias. Similarly, the tumor location was input as an image for the AI. Consequently, sex was included as a clinical feature in the AI learning process.

**Table 2:** Characteristics of patients with SCN and MCN. Values are presented as mean ± standard deviation or number. Age and size were tested using a Student's t-test. Sex, location, and vascularity were tested using a Fisher's exact test. MCN: mucinous cystic neoplasms; SCN: serous cystic neoplasms

Characteristics		SCN	MCN	p-value
Age, years (mean±SD)		52.2±4.5	46.3±8.1	0.64
Sex	Male	7	1	0.048
	Female	18	23	
Size (cm)		3.6±1.5	4.3±1.8	0.57
Location	Head	10	1	<0.01
	Body and tail	15	23	
Vascularity	Hyper	13	2	<0.01
	Iso-hypo	12	22	

Table 3 presents the indicators of experts, C-Single, and C-SingleCF. All AI results were significantly better than those of the experts. There was no statistically significant distinction between the modalities in C-Single. The accuracies of C-SingleCF were higher than those of C-Single. On EUS and T2, the accuracy of C-SingleCF was significantly greater than that of C-Single. Incorporating sex as a clinical feature enhanced the accuracy rate by approximately 6%.

**Table 3:** Three indicators of experts, C-Single, and C-SingleCF. C-Single: conventional single-modal artificial intelligence; C-SingleCF: C-Single with clinical features; EUS: endoscopic ultrasonography; ECECT: early phase of contrast-enhanced computed tomography; T2: T2-weighted magnetic resonance imaging; MRP: magnetic resonance pancreatography All models were tested using McNemar's test. *: All models demonstrated p<0.05 compared with experts. †: MI-Triple demonstrated a significantly higher accuracy compared to C-Single CF.  ‡: MI-Quad demonstrated a significantly higher accuracy compared to C-Single CF.

Model	Input	Sensitivity (%)	Specificity (%)	Accuracy (%)
Experts	EUS	68	78	76
	ECECT	64	68	68.8
	T2	64	75	70
	MRP	66	74	72
C-Single	EUS	76	87.5	81.6 * †
	ECECT	84	91.6	87.8 *
	T2	76	87.5	81.6 * ‡
	MRP	92	83.3	87.8 *
C-SingleCF	EUS+SEX	80	95.8	89.7 * †
	ECECT+SEX	88	95.8	91.8 *
	T2+SEX	84	91.6	89.7 * ‡
	MRP+SEX	96	87.5	91.8 *

For C-Single and C-SingleCF, the sensitivities of EUS, ECECT, and T2-weighted imaging were lower than their specificities. Conversely, the sensitivity of MRP exceeded its specificity. MRP exhibited distinct characteristics compared to the other imaging modalities.

Table 4 presents three indicators of experts who utilized all modalities and all combinations in MI-AI. All AI combinations outperformed experts significantly. When compared to C-SingleCF and MI-Double, adding one more modality to C-SingleCF enhances accuracy. MI-Triple and MI-Quad outperformed C-SingleCF significantly for all indicators.

**Table 4:** Sensitivity, specificity, and accuracy of experts and all combinations in MI-AI. C-SingleCF: conventional single-modal artificial Intelligence with clinical features, ECECT: early phase of contrast-enhanced computed tomography, EUS: Endoscopic ultrasonography, MI-AI: artificial Intelligence utilizing multimodal imaging, MI-Double: MI-AI employing double imaging modalities with clinical features, MI-Triple: MI-AI employing triple imaging modalities with clinical features, MI-Quad: MI-AI employing quad imaging modalities with clinical features, MRP: magnetic resonance pancreatography, T2: T2-weighted magnetic resonance imaging All models are tested by using McNemar's test. *: all models demonstrated p<0.05 compared with experts. †: MI-Triple demonstrated a significantly higher accuracy compared to C-Single CF. ‡: MI-Quad demonstrated a significantly higher accuracy compared to C-Single CF.

model	input	sensitivity (%)	specificity (%)	accuracy (%)
Experts	EUS+ECECT+T2+MRP+SEX	78	82	81
C-SingleCF	EUS+SEX	80	95.8	89.7*
	ECECT+SEX	88	95.8	91.8*
	T2+SEX	84	91.6	89.7*
	MRP+SEX	96	87.5	91.8*
	Average	85	93.7	91.8*†‡
MI-Double	ECECT+MRP+SEX	96	100	98.0*
	T2+MRP+SEX	100	95.8	95.9*
	EUS+MRP+SEX	96	95.8	95.9*
	ECECT+T2+SEX	96	91.7	93.9*
	EUS+T2+SEX	96	100	95.9*
	ECECT+EUS+SEX	88	87.5	91.8*
	Average	95.3	95.1	94.9*
MI-Triple	ECECT+T2+MRP+SEX	96	100	98.0*
	EUS+T2+MRP+SEX	96	100	98.0*
	EUS+T2+ECECT+SEX	96	100	98.0*
	EUS+ECECT+MRP+SEX	96	95.8	93.9*
	Average	96	99	97.0*†
MI-Quad	EUS+ECECT+T2+MRP+SEX	98	100	99.0*‡

## Discussion

EUS, CT, and MRI were utilized to categorize pancreatic disease. C-Single, using any of these techniques, has been documented in the classification of SCN and MCN [12-16]. These studies highlighted the efficacy of AI with a particular modality. However, a comparative analysis of these modalities has not been recorded.

In this study, we utilized T2, MRP, EUS, and ECECT for the AI classification. C-Single with each modality outperformed experts with statistical significance, but the difference between the C-Single modalities was not statistically significant. However, there were some differences in the properties of the modalities.

MRI has many sequences, and each hospital uses different sequences. The common MRI sequences are T1, T2, and MRP. MRI provides superior soft tissue contrast compared to CT, with T2 offering more advantages for differentiating cystic lesions than T1 and CT. This is because T2 clearly shows the lobulated contour or shape of these diseases [6, 13, 16]. Therefore, T2 was utilized in this study.

In general, MRCP offers crucial insights for clinical practice. For instance, pancreatic carcinomas often exhibit bile duct dilatation. However, in this study, we used MRP due to challenges in excluding the impact of cholecystectomy or gallbladder variations on MRCP. In C-Single, MRP demonstrated a distinct pattern compared to other imaging techniques. MRP exhibited high sensitivity and low specificity, while other modalities showed the opposite trends. MRP provides information on the entire pancreatic ductal system, encompassing cyst count, size, and the presence of ductal obstruction or dilatation. In contrast, alternative modalities only provide localized information regarding the tumor and its vicinity. The SCN's positioning significantly differed from that of the MCN, aligning with findings in other studies [13, 21, 24, 25]. This variance may elucidate the discrepancy between MRP and alternative modalities. MI-Double incorporating MRP outperformed C-SingleCF, highlighting the complementary nature of MRP and other imaging modalities.

In the C-Single analysis, although ECECT lacks information about the entire pancreatic duct system and has a lower detection rate for the honeycomb area [4], its performance indicators are comparable to those of other modalities. This can be attributed to the fact that ECECT provides information about tumor vascularity. Notably, 55% of SCNs are hypervascular tumors [4], while most MCNs are hypovascular. Therefore, ECECT is thought to be useful for distinguishing between hypervascular SCNs, MCNs, and hypovascular SCNs.

In the C-Single analysis, the results of EUS fell within the mid-range compared to the other three modalities. Although EUS does not provide a complete view of the entire pancreatic duct system or details on tumor vascularity, it demonstrates proficiency in identifying the honeycomb area [4]. Sun et al. reported enhanced diagnostic accuracy in distinguishing SCN from MCN using contrast-enhanced ultrasound [26]. Hence, the integration of contrast-enhanced EUS into AI models is expected to further enhance diagnostic accuracy. The indicators of C-Single and C-SingleCF were similar to the findings reported previously [12-16]. Incorporating additional test modalities enhances sensitivity and decreases outcome variance, as shown in a prior study [17-21]. The findings suggested that multimodal AI stabilized and enhanced the model's robustness.

In this study, multiple images obtained from several modalities were used. In another approach, differential diagnosis uses discrete values as a clinical feature instead of images from multiple modalities. Several studies have reported diagnoses based on imaging modalities and clinical features such as tumor vascularity, location, size, age, and sex [13]. Our results were higher than those reported in previous studies. It is assumed that the results were associated with the difference between the images and the discrete values of the clinical features. In these studies, tumor vascularity was described as a discrete value (hyper/not). Tumors have various vascularities and heterogeneities, and discrete values are too simple to express these features. It was difficult to set cut-off values for the two groups. Generally, previous studies classified tumor locations into two or three categories: head or body/tail or head, body, and tail. When a tumor was located on the border between two lesions or when tumors existed in multiple lesions, the tumor location was not described using this classification. Consequently, the use of discrete values or classifications results in a significant loss of information from the mesh. By contrast, AI can minimize information loss by learning directly from images. For this reason, MM-AI has higher accuracy than those studies. 

The sex and age were independent of the images. Normalizing images by tumor size allows the tumor size to be utilized as an independent parameter. Sex, age, and tumor size were described as discrete values. At this point, these are different from the vascularity and location of the tumor, as mentioned above. In this study, the sex differences in the SCN and MCN were statistically significant (Table 2). Therefore, we used sex as a clinical feature in this model. Table 3 shows that adding sex improves 4%-8% in accuracy to classify the two diseases. Every C-SingleCF was superior to C-Single, and two exhibited statistically significant results. Age and tumor size showed no significant differences in this study. Therefore, they were not used as parameters in the model.

AI using multimodal imaging demonstrated superiority over AI using single-modal imaging with clinical features. However, this study presents certain limitations and unresolved issues. One such limitation is the small number of participants in our cohort. In this study, we used clinical features that have statistical significance as parameters. Whether these clinical features are statistically different between the two diseases is controversial [13, 21, 24, 25]. There is a possibility that a significant difference will emerge in other factors. According to these factors, the AI model must be changed. 

One of the unresolved issues is the network and data fusion method. Most studies on medical image classification have used modified, already-proposed AI networks. However, a comparative evaluation of AI networks has not been performed, and an appropriate network for multimodal AI remains unknown. Moreover, the results or data of each modality must be fused in multimodal AI. Multimodal data fusion methods can be categorized into three types [22, 23]. Type-1 is feature-level fusing, in which the image is fused before deep learning; Type-2 is classifier-level fusing, in which the feature map is extracted and fused after deep learning, and Type-3 is decision-level fusing, in which it is fused after the last activation function at each layer. While we utilized a Type-2 model, it remains unknown whether this fusion approach is the optimal choice. Further investigation is required to identify the optimal fusion strategy for multimodal AI.

## Conclusions

In this study, AI utilizing multimodal imaging outperformed the experts and AI utilizing single-modal imaging. Future studies should explore the robustness of this model in a larger patient population and its application to a broader range of pancreatic tumors. While we utilized EUS, ECECT, T2, and MRP in this study, other phases of CT and additional MRI sequences have enhanced diagnostic accuracy. Although age and tumor size did not exhibit significant differences in this study, they could potentially prove effective when considering other pancreatic tumors.

While single-modal AI demonstrates ongoing improvements, its performance is progressively reaching a plateau. Consequently, despite several remaining challenges, research into multimodal AI is crucial.
